# Supplementary material for: Wet Synthesis of Elongated Hexagonal ZnO Microstructures for Applications as Photo-Piezoelectric Catalysts
Source: Materials (Basel). 2020 Jun 30;13(13):2938. doi: 10.3390/ma13132938 (PMC7372467; doi:10.3390/ma13132938)
Supplement: Supplementary file 1 [file materials-13-02938-s001.pdf]

materials

Supplementary Materials: Wet Synthesis of Elongated Hexagonal ZnO Microstructures for Applications as Photo-Piezoelectric Catalysts

Rosanna Pagano [1], Chiara Ingrosso [2], Gabriele Giancane [3,4,*], Ludovico Valli [1,4] and Simona Bettini [4,5]

[1] Department of Biological and Environmental Sciences and Technologies, DISTEBA, University of Salento, Via per Arnesano, Lecce I-73100, Italy; rosanna.pagano@unisalento.it (R.P.); ludovico.valli@unisalento.it (L.V.)
[2] CNR-IPCF Sez. Bari, c/o Department of Chemistry, University of Bari, via Orabona 4, Bari I-70126, Italy; c.ingrosso@ba.ipcf.cnr.it
[3] Department of Cultural Heritage, University of Salento, Via D. Birago, 48, Lecce I-73100, Italy
[4] Consorzio Interuniversitario Nazionale per la Scienza e Tecnologia dei Materiali, INSTM, Via G. Giusti, 9, Firenze I-50121, Italy; simona.bettini@unisalento.it
[5] Department of Engineering for Innovation, University of Salento, Via per Monteroni, Lecce I-73100, Italy
* Correspondence: gabriele.giancane@unisalento.it

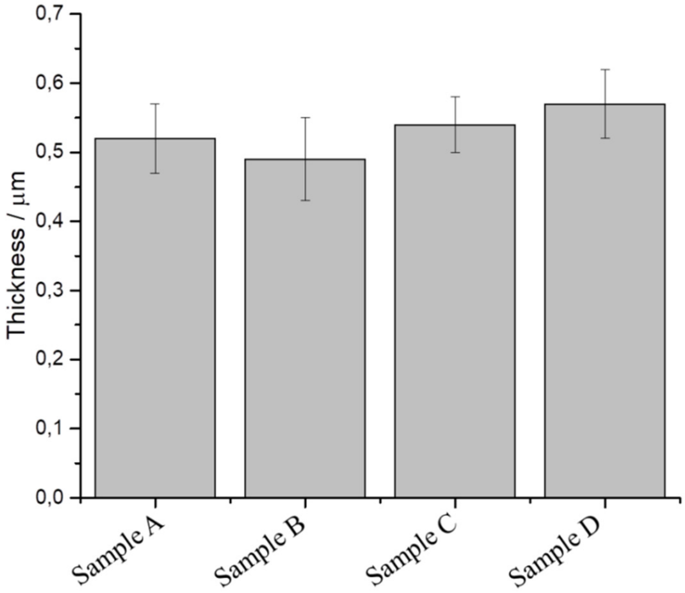

Figure S1. Thickness estimation of the samples A, B, C and D. The error bars were calculated as standard deviation of the results obtained repeating the measurement on 5 samples obtained in the same experimental conditions.

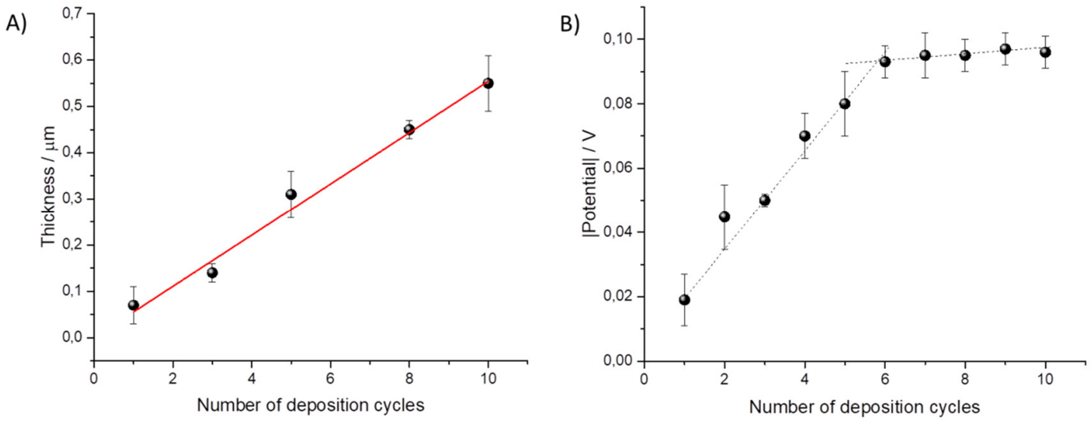

Figure S2. A) Thickness estimation of ZnO film as a function of different number of deposition cycles. **B**) Piezo-potential recorded for samples with different thickness. An asymptotic value can be reached when a thickness of the piezoelectric layers approaches about 0.35 µm.

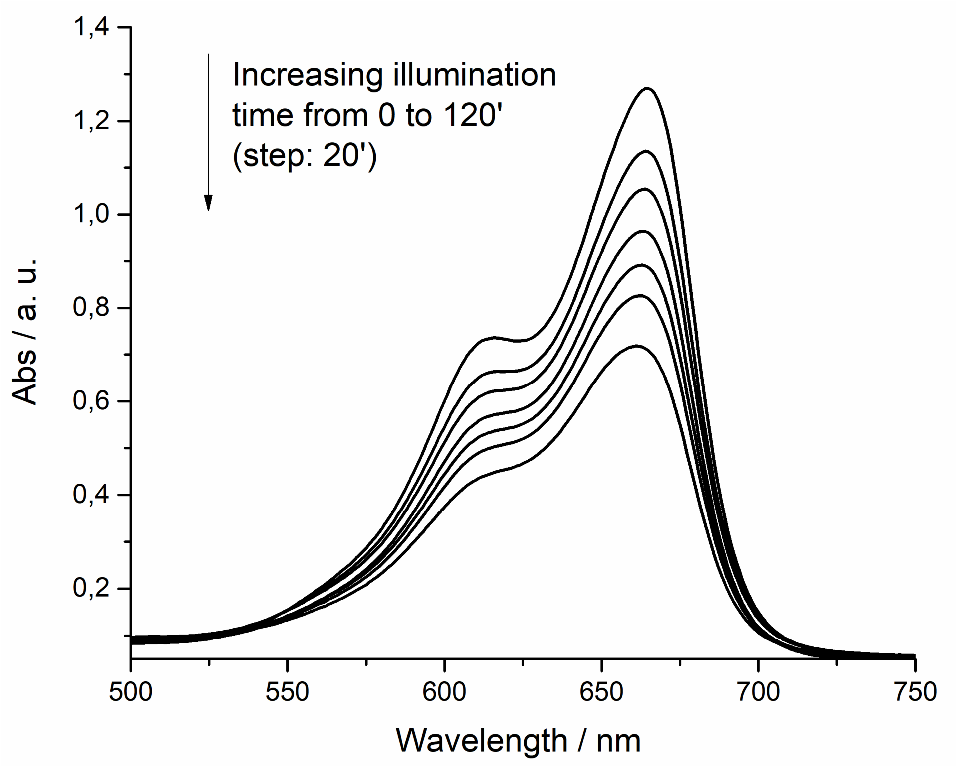

Figure S3. Photo-degradation of methylene blue assisted by ZnO powder of sample A.

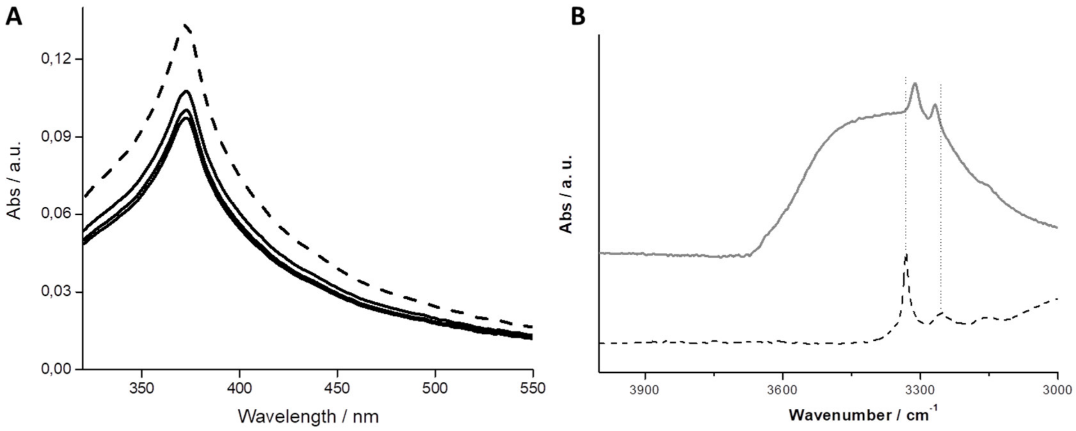

Figure S4. A. Bleaching of the ZnO absorption peak before the substrate immersion in water (dotted line) and after 10, 20 and 30 minutes immersion (continuous lines). **B**. FTIR spectra at high wavenumber (4000-3000 cm^{-1}) of ODA thin film and ZnO/ODA multilayer.

© 2020 by the authors. Submitted for possible open access publication under the terms and conditions of the Creative Commons Attribution (CC BY) license (http://creativecommons.org/licenses/by/4.0/).
